# Scalability Analysis of LoRa and Sigfox in Congested Environment and Calculation of Optimum Number of Nodes

**DOI:** 10.3390/s24206673

**Published:** 2024-10-17

**Authors:** Mandeep Malik, Ashwin Kothari, Rashmi Pandhare

**Affiliations:** 1 Indian Institute of Information Technology (IIIT), Nagpur 441108, India; rpandhare@iiitn.ac.in; 2 Visvesvaraya National Institute of Technology (VNIT), Nagpur 440010, India; ashwinkothari@ece.vnit.ac.in

**Keywords:** LoRa, Sigfox, Packet Error Rate, LPWAN

## Abstract

Low-power wide area network (LPWAN) technologies as part of IoT are gaining a lot of attention as they provide affordable communication over large areas. LoRa and Sigfox as part of LPWAN have emerged as highly effective and promising non-3GPP unlicensed band IoT technologies while challenging the supremacy of cellular technologies for machine-to-machine-(M2M)-based use cases. This paper presents the design goals of LoRa and Sigfox while throwing light on their suitability in congested environments. A practical traffic generator of both LoRa and Sigfox is introduced and further interpolated for understanding simultaneous operation of 100 to 10,000 such nodes in close vicinity while establishing deep understanding on effects of collision, re-transmissions, and link behaviour. Previous work in this field have overlooked simultaneous deployment, collision issues, effects of re-transmission, and propagation profile while arriving at a number of successful receptions. This work uses packet error rate (PER) and delivery ratio, which are correct metrics to calculate successful transmissions. The obtained results show that a maximum of 100 LoRa and 200 Sigfox nodes can be deployed in a fixed transmission use case over an area of up to 1 km. As part of the future scope, solutions have been suggested to increase the effectiveness of LoRa and Sigfox networks.

## 1. Introduction

The internet of things (IoT) covers a large ambit of possible technologies that work in different spheres of communication and present need-based solutions [1]. It also creates multiple opportunities to streamline connectivity issues for wide-ranging use cases as per requirement. Data generated by billions of these low-cost ultra-low-power sensors are available on the cloud for big data analytics, and the efficiency of these sensors can be further increased by the application of machine learning (ML) algorithms [2,3]. IoT-based sensors will be acting as our eyes, ears, and noses as they are implemented for capturing data related to temperature, humidity, air quality, and location and thus become building blocks for use cases such as smart metering, home automation, precision lighting, smart parking, and other day-to-day applications to mitigate global challenges like energy crises, natural disasters, and numerous others [4,5]. IoT is a flexible network that supports many wide-ranging communication options from a few meters to a few miles and is also available through licensed and unlicensed bands, providing multiple connectivity options. LPWAN-based technologies complement IoT with communication ranges of up to 15 km, enabling the wide area communication pillar [6]. According to [7,8] there are currently 30 billion IoT devices and are expected to reach 75 billion by 2025, all of which will be contesting within unlicensed spectrum to transmit data in smart cities [9]. LPWAN technologies as part of IoT also present an alternative to cellular networks as they provide wide area communication unlike current short-range IoT techniques like Bluetooth, WiFi, and Zigbee [10,11]. Two of the most prominent LPWAN technologies are Long Range (LoRa) and Sigfox. LoRa is a WAN technology for ultra-low-power devices communicating over large distances on low bandwidth using unlicensed bands. LoRa uses chirp spread spectrum (CSS), which provides strong resistance against noise, interference, and multipath fading effects. LoRa and Sigfox provide a platform for numerous widespread use cases such as smart agriculture, positioning, wearable, and other smart city applications replacing conventional mobile networks like 2G, 3G, 4G, and 5G, which consume higher energy and are capital intensive due to higher spectrum costs [12]. LoRa, developed by Semtech, uses the CSS technique to spread a narrow band signal over the full channel bandwidth, while Sigfox uses differential binary phase-shift keying (DBPSK) in industrial, scientific, and medical (ISM) radio bands. Both techniques increase the distance of signal while reducing power consumption as they are narrow band and thus sacrifice on data rate to enable larger distances with longer battery life [13,14].

**Figure 1:**
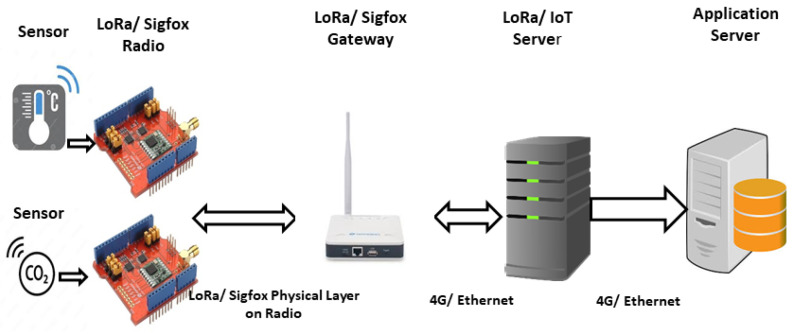
Deployment Architecture of LoRa and Sigfox.

LoRa uses LoRAWAN at the medium access control (MAC) layer, which makes use of ALOHA as a random access technique, while Sigfox uses random frequency time division multiple access (RFTDMA) for channel access. Both techniques transmit in a random and uncoordinated fashion, thus any increase in the number of IoT devices will lead to an exponential increase in collisions. LoRa and Sigfox technologies promote fair usage of ISM band [15]. Sigfox is both the manufacturer and network operator for its devices [16]; its network is currently deployed in Europe. LoRa, on the other hand, has been deployed across continents as the MAC of LoRa can be operated by telecommunication service providers (TSP). Multiple configurable parameters of LoRa, such as spreading factor (SF), bandwidth (BW), coding rate (CR), transmission power (TP), and message length, can be tweaked in order to decrease the packet error rate (PER), thus providing a reliable communication environment for successful transmission. Figure 1 depicts the deployment of LoRa and Sigfox, wherein sensors are connected to LoRa/Sigfox nodes and sensor data are transmitted to the LoRa/Sigfox gateway through the radio network. Since LoRa/Sigfox are not just connected to a single gateway, multiple gateways are able to receive this communication, which is then forwarded to the LoRa server maintained by the telecommunications company, or LoRaWAN. At this point, duplicate copies of the same message are filtered out and finally passed to the application server maintained by the network provider. LoRa and Sigfox are excellent networks when any kind of low-data-rate applications are envisaged since they carry flexibility coupled with a small form factor and long battery life. Deployment as carried by any telecommunication service provider (TSP) will require the control of the IoT server or application server for the generation of downlink control packets to streamline the number of messages being transmitted by each device; this will reduce the number of collisions by updating each device with current network situation awareness.

In this paper, we consider dense deployment of LoRa and Sigfox for smart city applications where millions of such devices will be contesting to transmit within a limited BW. This enables understanding the scalability of LoRa and Sigfox to calculate collisions. Our motivation is the study of LoRa and Sigfox to evaluate LoRa link behaviour with multiple SF and fixed bandwidth of 250 KHz and 100 Hz for LoRa and Sigfox, respectively. We practically examine the link behaviour of single LoRa and Sigfox nodes through outdoor experimentation and then use the same data for traffic generators of Sigfox and LoRa to simulate collisions and successful transmissions. We also calculate the number of collisions as we increase/decrease SF; we also use PER and delivery ratio to obtain the number of devices that can coexist in an outdoor environment with fixed transmissions and BW. In the end, we simulate the effects of random access, re-transmissions, path loss, and time on air (ToA) while using the propagation model with urban area settings.

## 2. Related Work

Simulating LoRa devices in a real-world (smart city) scenario is very difficult, as the effects of screening, thermal noise, large-scale fading, and small-scale fading are random in nature and contribute in a significant manner towards collisions, re-transmissions, and further collisions as the number of devices is increased. Ref. [17] in 2016 estimated that a maximum of 120 devices can coexist simultaneously in a given area based on a practical matrix called data extraction rate (DER). The work considered LoRa to have imperfect orthogonality and did not consider re-transmission success. Ref. [18] published on LoRa scalability used packet delivery rate (PDR) and bit error rate (BER) to determine success of transmission; however, Ref. [19] described packet error rate (PER) as the factual criteria for determining success but did not take re-transmissions into consideration; Ref. [20] worked on a dense urban scenario of Sigfox nodes based on the signal-to-noise ratio (SNR) and BER to measure success probability. Work in [21] carried out extensive trials over a campus with LoRa while studying different propagation models. Authors in [22] used NS3 and Matlab to carry out simulation for 100 devices with faster run-time in comparison to previous work. In [23,24], work was carried on for city-based use cases along with gateway location while using the Ericsson propagation model. Reef. [19] presented experimental work on a number of Sigfox devices that can coexist without collision in a smart city environment, while the same was substantiated by [25] and described PDR as correct matrices for determining successful delivery of transmission. PDR can be calculated as the ratio of the total number of up-link messages to received messages. In [25], the author also established that PDR decreases as distance increases and that LoRa gives a higher performance in terms of PDR in comparison to Sigfox, however work used probability of outage as major criteria for deriving packet loss and did not consider dense deployment and thus arrived at a lower PER/PDR value. Previous works have not considered the effects of path loss in urban environments and have not carried out practical comparisons of LoRa and Sigfox to arrive at a number of nodes that can coexist.

## 3. LPWAN Technologies and Design Goals

LPWAN technologies have the ability to communicate at long range using low power and provide cost effective connectivity to a massive number of IoT devices. Common design goals for LPWAN devices are high reliability, long range, low cost, small form factor, and long battery life. Leading LPWAN [26] technologies are LoRa [15], Sigfox [16], systems offered by manufacturers TELENSA [27], INGENU [28], QOWISIO and standards [29], WEIGHTLESS-N [30], and WEIGHTLESS-P [31]. Among them, TELENSA, QOWISO, LoRa, and Sigfox work in the sub-GHz band unlike others and thus exploit better propagation properties and immunity to noise for a wider coverage area [32,33]. The primary design goal for all LPWAN devices is to keep the end device simple, carry out limited transmission, use simple protocols, remain low cost, provide high sensitivity, and have deep sleep capability when not transmitting. These design goals also lead to uncoordinated and random communication, which is a major limitation of LoRa and Sigfox and their deployment in dense environments. Technical details of Sigfox and LoRa for commercial deployment are given in Table 1, and their design goals are given in succeeding paras.

### 3.1. Radio Spectrum and Modulation

LPWAN technologies, as subsets of IoT, provide wide coverage for outdoor communication. Previously mentioned systems work in the sub-GHz band to utilise excellent signal propagation properties of said band. LPWAN technologies are also designed to operate with a low power budget and at up to 20% higher sensitivity to connect with the base station while mitigating the effects of the multi-path fading and attenuation experienced in higher bands. Receiver sensitivity for LPWAN devices is typically planned around −130 dB with narrow-band and spread spectrum options. The narrow-band technique maximizes the range by assigning narrow-band channels to each carrier, thus reducing noise effects and also reducing the processing required for decoding the signal and thus promoting inexpensive receivers. NB-IoT [34] and WEIGHTLESS-P [35] are perfect examples of narrowband techniques, while Sigfox, WEIGHTLESS-N, and TELSENA [27] are examples of ultra narrow band (UNB) communication with 100 Hz bandwidth enabling the possibility of more end devices and reduced interference. Spread spectrum techniques on the other side will require higher processing to decode, since the signal is spread over a wider frequency and is below the noise floor while being more resilient to interference and jamming.

### 3.2. Link Budget

LoRa and Sigfox work on a tight link budget of 150±10 dB [36], which lets them attain a coverage of up to 15 km in a rural area and a few kilometres in an urban environment. In order to attain a high sensitivity of up to −130 dBm [15], LoRa sacrifices the data rate by decreasing the modulation rate to place more energy in each symbol, enabling the reception of highly attenuated signals. LoRa uses the spread spectrum modulation technique for adding noise immunity; Sigfox, on the other hand, uses the ultra narrow band (UNB) technique [16]. LoRa receiver sensitivity will essentially depend on the spreading factor, which typically ranges between −130 dBm and −105 dBm; the same can be calculated for a LoRa device with a transmitting power of 14 dBm, distance *d* as 1 km, a path loss exponent (PLE) of n=3 for urban area setting, path loss reference distance $d_{0}$ (1 m) of 32 dB, and a nominal antenna gain of 2 dBi. The path loss and link budget are calculated in dB for the above data and given in Equations (1)–(3).

(1) $$Pathloss=10*n*log_{10}\left(d\right)+L_{0}$$

(2) $$Pathloss=10*3*log_{10}\left(1000\right)+32=92$$

(3) Linkbudget=14+2+2−92=−74

Thus this value of link budget is well below the receiver threshold of −130 dBm, also use of higher spreading rate will increase receiver sensitivity.

### 3.3. Topology and Scalability

LoRa and Sigfox both use star topology for forwarding messages to gateways as being used by cellular networks, and end devices are hooked to a single gateway; however, their transmission may be received by multiple nearby gateways. Key requirement for LPWAN devices is scalability while working on low volumes of traffic and still coping with increasing density of devices by making use of more number of gateway controllers and adaptive data rate for collision-free communication. In order to increase scalability, LoRa configures multiple communication parameters to increase or decrease data rate and ToA. Mesh and tree topology is also used by some technologies, but with limited adaptation as the same will require complex protocols and higher consumption of battery, which are in contravention of the design goals of LPWAN. LoRa and Sigfox are meant for machine-to-machine communication on lines of cellular networks to support an IoT grid for sensors while keeping the capital expenses (CAPEX) and operation expenses (OPEX) to minimum.

### 3.4. LoRa Duty Cycling

Duty cycling is heavily used in LPWAN devices to turn off the power-hungry transceiver through pre-arranged listening and transmission schedules [37]; duty cycling is used at the rate of 1% i.e., 36 s per hour. Duty cycling is also carried out due to limited spectrum availability, where devices have to coexist within a limited unlicensed band. Duty cycle limitation for Sigfox devices is around 0.1% to 1%, similar to LoRa. The duty cycle is guided by government and regional regulations; for instance, in Europe, under the regulations of the European Telecommunications Standards Institute (ETSI), limitation in the 868 MHz band is 1%. LoRa is more versatile and flexible, while Sigfox is a more stringent and simpler transmission technology while supporting applications with fixed data rates, such as smart metering and others. Duty cycling for Sigfox can be calculated using the formula as given in Equation (4).

(4) $$D=\frac{T_{ON}}{T_{Total}}*100\%$$

where *D* represents duty cycle, $T_{ON}$ is total transmission time, and $T_{TOTAL}$ is total time in a day. Now, each transmission of a Sigfox device is of 6 s and taking the device transmits the maximum number of messages in a day, i.e., 140 messages per day, $T_{ON}$ will be 140 messages × 6 s per message, calculated as 840 s and $T_{TOTAL}$ will be 86,400 s for a 24 h cycle. Thus, the duty cycle for a Sigfox device $D_{Sigfox}$ can be calculated as following:

(5) $$D_{Sigfox}=\frac{840}{86400}*100\%=0.97\%$$

### 3.5. Contention Mechanism

LPWAN devices use lightweight and simple MAC protocols to conserve power. LoRa and Sigfox are based on the ALOHA protocol and do not utilize carrier sense multiple access collision avoidance or collision detection (CSMA CA/CD) mechanism as being used in other wireless technologies. Also, as both work in unlicensed bands, the number of devices is not controlled, thus CSMA may become ineffective due to the increase in the number of devices.

## 4. System Model

### 4.1. LoRa System Model

In order to carry out the link-level study of LoRa and Sigfox, it is necessary to understand the configurable communication parameters open to optimization, such as spreading factor (SF), transmission power (TP), bandwidth (BW), coding rate (CR), and payload (PL). LoRa uses CSS [38] for modulation, where an up-chirp ranges between $\left(f_{o},f_{1}\right)$ and a down-chirp between $\left(f_{1},f_{o}\right)$, thus the BW can be calculated as $BW=f_{1}-f_{o}$, and *n* the LoRa symbol is expressed in Equations (6) and (7).

(6) $$f\left(t\right)=\{f_{1}+k\left(t-n.T_{chip}\right)for\left\{0\leq t\leq n.T_{chip}\right\}$$

(7) $$f\left(t\right)=\{f_{0}+k\left(t-n.T_{chip}\right)for\left\{n.T_{chip}\leq t\leq T\right\}$$

where slope is described as $k=(f_{1}-f_{0})/T$ and total number of symbols are dependent upon SF, given as $2^{SF}$. Symbol transmission time is $T_{sym}=2^{SF}/BW$, length of one symbol is given as $t_{s}=SF/B$, and bit rate is calculated as $R_{b}\left(SF\right)=log_{2}\left(SF\right)/t_{s}$. LoRa packet format contains preamble, physical header, PL, and cyclic redundancy check (CRC). Once a frame is transmitted, LoRa opens a reception window to receive acknowledgement. The time for the first window of reception is the same as the transmit time, and the time for the second window is the same for all LoRa devices. The total time taken by a frame to pass is the sum of PL and preamble time and can be represented as $T_{PKT}=T_{PRE}+T_{PAY}$ where $T_{PKT}$ is the total time for packet to get transmitted and $T_{PRE}$ and $T_{PAY}$ [39] are the time taken by preamble and payload, respectively. Time to transmit preamble is given as $T_{PRE}=\left(N_{PRE}+4.25\right)T_{SM}$, which can be further described as $T_{PRE}=\left(N_{PRE}+4.25\right)\frac{2^{SF}}{BW}$, while payload is given as $T_{PAY}=\left(N_{PL}*T_{SM}\right)$. As described in [40], preamble can be programmed as per use case, thus a temporary variable TEMP is used to define the packet composition and its time on air (ToA) for onward simulations. Variable TEMP is defined as following.

(8) $$TEMP=\frac{28+8PL+16CRC-4SF-20H}{4(SF-2DE)}$$

Now, payload can be given as

(9) $$N_{PL}=8+\max\left(ceil\left[TEMP\right]\right)\left(CR+4\right)),0$$

Transmission time of payload $T_{PAY}$ can be obtained as

(10) $$N_{PL}=8+\max\left(ceil\left(\frac{8PL-4SF+28-20H}{4(SF-2DE)}\right)\left(CR+4\right)\right)$$

(11) $$N_{PL}=8+\max\left(ceil\left[TEMP\right]\right)\left(CR+4\right))\frac{2^{SF}}{BW}$$

Finally, the total packet transmission time $T_{PKT}$ is given as

(12) $$T_{PKT}=(N_{PRE}+4.25+\left(8+max\left[ceil\left[TEMP\right]\left(CR+4\right),0\right]\right)\frac{2^{SF}}{BW}$$

(13) $$T_{PKT}=(N_{PRE}+4.25+\left(8+max\left[ceil\frac{28+8PL+16CRC-4SF-20H}{4(SF-2DE)}\left(CR+4\right),0\right]\right)\frac{2^{SF}}{BW}$$

where PL is in bytes, SF is from 7 to 12, cyclic redundancy check CRC has options of ‘1’ and ‘0’, *H* is the header mode with values of ‘0’ or ‘1’, and data rate optimization for long payloads DE represents data optimization if used. LoRa provides three different bandwidth options of 125 KHz, 250 KHz, and 500 KHz within the allotted spectrum, and the chip rate is decided by bandwidth. A larger bandwidth enables a better quality signal, as it is more resilient to multipath fading. LoRaWAN abstracts over the SF and bandwidth to provide a parameter called dataratem; the airtime of LoRa depends on SF, BW, CR, and PL and can have a significant effect on collisions. SF can change the ToA of 20 byte LoRa packet from 9 ms to 1.3 s. Possible data rates as available through different SF and SNR values for LoRa are shown in Table 2. The capacity of LoRa ($C_{lora}$) can be calculated using Table 2 as following.

$$C_{lora}=1*\left(SF\left(7-12\right)\right)=1*\left(293,537,976,1757,3125,5468\right)bytes/s=12.156kb/s$$

LoRa makes use of simple linear block code such as hamming code for forward error correction (FEC), which is easy to implement and offers coding blocks with minimum hamming distance of 1, 2, 3, and 4 for code rates 4/5, 4/6, 4/7, and 4/8, respectively. A CR can increase the ToA as more number of bits are utilized while giving error detection and correction capabilities. CR of 4/8 means that four bits are being encoded by eight bits, while the sensitivity of the receiver can be increased by using a CR of 4/8 though link budget and ToA will also increase. The CR, SF, and data rate relationship is given in Equation (14).

(14) $$R_{b}=SF.\frac{B}{2^{SF}}.\frac{4}{4+CR}$$

### 4.2. System Model: Sigfox

Sigfox is deployed over a BW of 192 KHz with a channel BW of 100 Hz; maximum equivalent radiated power (ERP) of a device is 14 dBm or 25 mW [41]. A node can only transmit 140 messages a day with maximum size of 12 bytes over a data rate of 100 bits/s [23]. Sigfox frame size is 26 bytes, and it uses spatial diversity in architecture as each message is transmitted three times with different frequency. Downlink messages are capped at four messages per day of 8 byte length, limiting the implementation of acknowledgements and optimization schemes. At a physical layer, Sigfox uses modulation schemes, as given in Table 1; Also, there is no synchronization mechanism between the transmitter and receiver, which helps in the reduction of complexity and cost. Sigfox uses BPSK for uplink and GFSK for downlink; random frequency time division multiple access (RFTDMA) is used for multiple access. If we consider payload of 12 byte, then there will be 6 messages that will be transmitted in one hour, each taking close to 2.08 s. We calculated the number of channels available to Sigfox, where each channel is of 100 Hz and the total bandwidth allocated to Sigfox is 192 KHz within the ISM band, i.e.,  SRD868 in Europe and ISM 900 in the USA. There are a total of 1920 channels available for transmission, though Sigfox uses spatial diversity where each message is modulated with three different frequencies and transmitted at three different time intervals limiting its effective transmission capability. It is highlighted that if orthogonality is applied to 1920 channels of Sigfox, only 360 channels are available for transmission, and due to the timing offset, collision chances increase. Sigfox node and gateway have been implemented using a Raspberry Pi single-board computer with an Arduino MKR 1200 board, a Sigfox SDR dongle, SMA connectors, and Lime SDR using GNU radio as gateway. Device used are as per zone 1 (RC1) configuration, which is applicable in Asia, and the module is UPLYNX Sigfox and TD1207. Path loss is calculated using received signal strength indicator (RSSI) and SNR as $PL=\left|RSSI\right|+SNR+P_{TX}+G_{RX}+10n\log_{10}\left(\frac{d}{d_{0}}\right)$. Where PL is path loss, $P_{TX}$ is ERP, $G_{RX}$ is receiver gain, *n* is path loss component, *d* is distance between Sigfox device and gateway, and $d_{0}$ is reference distance. Sigfox dataflow can be derived using the following Algorithm 1 for transmission and Algorithm 2 for collision calculation in case of LoRa.
**Algorithm 1** Sigfox Traffic FlowStep 1: Start with data generation using Sigfox stack library Step 2: GNU frame import as per Sigfox frame format Step 3: Initiate Lime SDR, physical layer signalling Step 4: Transmit data over radio interface Step 5: Repeat steps 2 to 4 with t second offset

**Algorithm 2** Collision and PER CalculationStep 1: Input: *n* nodes, *k* gateways with *A* area Step 2: Define interval $T_{s}$, SF 7-12, frequency f=868.7MHz Step 3: Find distance from *k* to each *n*; set BW=250KHz Step 4: Calculate path loss (Hata) *L*, ${LR}_{n,k}$ and SNR Step 5: Define interfering node *i* for all *n* in *A* Step 6: Duty cycle $D_{n,\beta_{s}}$ and packet arrival rate λ Step 7: For n,k, calculate *i* for each node Step 8: For j=1…m, calculate $P_{C}$, $P_{O}$, $D_{n}$ Return: $P_{C}$ and PER

## 5. Collision Calculation Through Packet Error Rate

Path loss model used for simulation in this paper is Hata Model [42] with correction factor of urban environment (9 dB) and described as follows:

(15) $$L=26.1\log_{10}\left(f\right)-13.8\log_{10}\left(h_{B}\right)-3.2{\left(\log_{10}\left(11.5h_{M}\right)\right)}^{2}-4.9+\left[44.9-\log_{10}\left(h_{B}\right)\right]log_{10}\left(d\right)$$

where *L* is the path loss, *d* represents the distance between transmitter and receiver, *f* is the frequency of operation $h_{B}$ is the height of base station and $h_{M}$ is the height of the LoRa device. The sensitivity of LoRa is given in Equation (16).

(16) $$S=-174+10\log_{10}BW+NF+SNR$$

Equation (15) follows the long-distance path loss model [42] of radio propagation, and thus the received power of an individual node *n* at gateway *k* is given by Equation (17), which presents the long-distance path loss model used to estimate signal attenuation as a function of distance, helping with network planning, link budgeting, predicting signal strength, and interference management.

(17) $$LR_{n,k}^{r}=P_{n}^{t}-\bar{PL}\left(d_{0}\right)-10\gamma \log\left(\frac{d_{n,k}}{d_{0}}\right)-Y_{\sigma }$$

where $P_{n}^{t}$ is the transmitted power, while the mean path loss is represented as $\bar{PL}\left(d_{0}\right)$, $d_{0}$ is the reference distance for the calculation of path loss, and $d_{n,k}$ defines the distance from node *n* and gateway *k*. $Y_{\sigma }=N\left(0,\sigma \right)$ represents a random variable with a Gaussian distribution, while σ is standard deviation with mean 0. Packet generation is considered to follow Poisson distribution. The probability of each LoRa station represented as *n* transmitting *l* packets over a period *T* is $c\left(l\right)=\frac{{\left(\phi T\right)}^{l}e^{-\phi T}}{l!}$, where ϕ is the message sending rate of LoRa; thus, the non-transmission probability of LoRa will be $Q\left(l=0\right)=\overset{N}{\underset{i=1}{∐}}e^{-\phi T}=e^{-\phi TN}$. LoRa uses ALOHA for transmission and has quasi-orthogonality [43] to different SFs by means of capture effect [44], which enables successful reception in case of overlapping transmissions with 6 dB difference, i.e., same SF and different SF. The reception at the LoRa gateway is decoded on the basis of a threshold SNR and interference value. The delivery ratio $D_{n}$ of a node *n* transmitting to *k* gateways will depend on the number of *n* transmitting devices in that window with same SF and the distance of such nodes from each other and gateway *k*. Successful reception will also be a function of ToA ($T_{s}$) and interfering LoRa devices also transmitting with same SF represented as $IF_{n.SF}$ [44] and given as Equation (18).

(18) $$D_{n}=\overset{12}{\underset{SF=7}{∐}}e^{-\phi T_{s}IF_{n.SF}}=\exp\left(-\phi \sum_{SF=7}^{12}T_{s}.IF_{n.SF}\right)$$

Packet transmission rate is $\phi \beta_{s}$, where $\beta_{s}$ is packet generation rate following a Poisson distribution and defined as $\beta_{s}=1-100\left(1-\beta \right)\phi T_{s}$. Delivery ratio incorporating effects of duty cycling can be further improved as given in Equation (19).

(19) $$D_{n,\beta_{s}}=\exp\left(-\phi \sum_{SF=7}^{12}T_{s}.IF_{n.SF}\beta_{s}\right)$$

LoRa gateways work in a star of stars, and devices are not associated with any single gateway, thus transmissions are received by multiple gateways, which forward messages to the network server for weeding out duplicate messages. A collision occurs when two or more than two LoRa devices transmit and generate interference, taking that at least one node transmits $N_{n}^{1,2}$ and generates no interference event denoted as $C_{N_{n}^{1,2}}$, its complement will be case of collision given as $C_{N_{n}^{1,2}}^{\prime }$, probability of such event will be $P(C_{N_{n}^{1,2}}^{\prime })$ and further given in Equation (20), also describing probability of a complementary event in a communication system, involving collision avoidance or successful transmission in a multi-node network.

(20) $$P\left(C_{N_{n}^{1,2}}^{\prime }\right)=1-P\left(C_{N_{n}^{1,2}}^{\prime }\right)=1-e^{\phi G(N_{n}^{1,2})}$$

Also considering that at least one LoRa device is transmitting in areas of gateway 1, $N_{n}^{1}$, and gateway number 2, $N_{n}^{2}$, probability can be represented as follows:

(21) $$P\left(C_{N_{n}^{1}}^{\prime }\cap C_{N_{n}^{2}}^{\prime }\right)=\left(1-P\left(C_{N_{n}^{1}}^{\prime }\right)\right)\left(1-P\left(C_{N_{n}^{2}}^{\prime }\right)\right)=\left(1-e^{\phi G(N_{n}^{1})}\right)\left(1-e^{\phi G(N_{n}^{2})}\right)$$

Now, the delivery ratio incorporating both events and also considering probability of regions from where LoRa devices are transmitting, same can be deduced as given in Equation (22).

(22) $$D_{n}=1-P\left(C_{N_{n}^{1,2}}^{\prime }\cup \left(C_{N_{n}^{1}}^{\prime }\cap C_{N_{n}^{2}}^{\prime }\right)\right)=e^{-\phi G(N_{n}^{1,2})}.[e^{-\phi G(N_{n}^{1})}+e^{-\phi G(N_{n}^{2})}-e^{-\phi G(N_{n}^{1}\cup N_{n}^{1})}]$$

In order to estimate collisions in a smart city environment with multiple gateways, regions, and hundreds of LoRa devices, let us consider a set $N_{n}^{1,2}$ covering a region of two gateways, $N_{n}^{1}$, and $N_{n}^{2}$. Let the area covered be given as *A*, thus the successful reception of packets based on De Morgan’s law will be given as following.

(23) $$PER=1-P\left(\overset{\left|A\right|}{\underset{i=1}{\cap }}\left(\underset{N_{n}^{k}\in A_{i}}{\cap }C_{N_{n}^{k}}^{\prime }\right)\right)=\prod_{i=1}^{\left|A\right|}1-\prod_{N_{n}^{k}\in A_{i}}(1-e^{\phi G(N_{n}^{k})})$$

Applying long distance path loss as given in Equations (15) and (17) and a set of interfering nodes represented by $I_{n}^{k},\forall k\in \left\{1,2,\ldots k\right\}$, Equations (22) and (23) will be same for value of k=1, path loss given in Equation (17) for a node *n* and a LoRa device transmitting at same time represented as *j* is $K_{i}^{r}=a_{i}+Y_{i}$ where i∈{n,j} and $Y_{i}=N\left(0,\sigma \neq 0\right)$. For successful reception, power at LoRa device *n* should be greater than power at interfering device *j*, i.e.,  $Pwr_{n}^{r}-Pwr_{j}^{r}<{SIR}_{s,s^{\prime }}$. Simplifying n=1, j=2 and ${SIR}_{s,s^{\prime }}$ = *b*, taking zero mean normal deviation $X_{1}-X_{2}<b-\left(a_{1}-a_{2}\right)$. The probability density function (PDF) will be $f\left(X_{3}\right)=\frac{1}{2\sigma \sqrt{2\pi }}e^{-\frac{x\frac{2}{3}}{8\sigma^{2}}}$. Outage probability is also affected by the fading environment, and thus the final delivery rate for LoRa device *n* will be $D_{n}=\left(1-P_{C}\right)\left(1-P_{O}\right)$, where $P_{C}$ is the probability of collision and $P_{O}$ is probability of outage. Based on the a/m scenario, $P_{C}$ and $P_{O}$ are presented in Equation (24) and Equation (25), respectively.

(24) $$P_{C}=\prod_{i=1}^{\left|s\right|}(1-(\prod_{R_{n}^{k}}\in s_{i}(1-\prod_{j\in R_{n}^{k}}P(C_{\left\{j\right\}}^{\prime }\cap C_{j}^{k}))))$$

(25) $$P_{O}=\frac{1}{2}\left[erf\left(\frac{P_{n}^{t}-P_{s}-\bar{PL}\left(d_{0}\right)-10\gamma \log\left(\frac{d_{n}}{d_{0}}\right)}{\sigma \sqrt{2}}\right)+1\right]$$

Probability of collision $P_{C}$ guides on the number of collisions that might occur in the *n* LoRa device scenario with *k* gateways, where *j* LoRa devices are considered interfering devices; $P_{O}$ gives guidance on the probability of the received power at the LoRa node and also the sensitivity for successful reception. Together, both are useful in determining successful reception and complete loss of packets. We can rewrite the equation for delivery ratio as follows:

(26) $$D_{n}=\left(1-P_{C}\right)\left(1-\prod_{k\in M}P_{O}^{k}\right)$$

Beyond the value of delivery ratio $D_{n}$, outage probability $P_{O}$, and collision probability $P_{C}$, the calculation of the re-transmission ratio (RT) based upon number of LoRa packets re-transmitted after collision will guide on the success of Algorithm 2.

(27) $$RT=1-\prod_{j\in IN_{n}}(1-P\left(C_{\left\{j\right\}}^{\prime }P\left(C_{j}\right)\right)$$

## 6. Simulation Results and Discussion

This section evaluates the number of LoRa/Sigfox devices that can coexist together in congested deployment and astutely estimates the number of nodes that can successfully transmit mitigating collision effects while reducing PER/re-transmissions and increasing the delivery ratio, which represents the effectiveness of transmission.

In the Europe region, LoRa has three 125/250 KHz channels (i.e., 868.10, 868.30, and 868.50 MHz) as part of the EU 863–870 MHz band [15]. In terms of transmitted power, the maximum is 14 dBm (25 mW) with multiple options such as 11 dBm, 8 dBm, 5 dBm, and 2 dBm. Incorporating these limitations, LoRa still has potential as it takes advantage of orthogonality among different SF and sends multiple packets simultaneously.

In order to conduct outdoor experimentation for generating data for a traffic generator, six LoRa SX1276 LoRa shields and six Arduino UNOs were programmed for transmission on same and different SFs along with two single-channel gateways. SX1276 works at 868 MHz, though less noise resistant in comparison to SX1278 working at 433 MHz. SX 1276 was chosen to draw a close comparison to Sigfox TD1207 (868 MHz). The Dragino single-channel gateway was placed on the rooftop of a seven-storey building (32 m), and LoRa devices were placed on buildings ranging from 7 m to 22 m in height.

**Figure 2:**
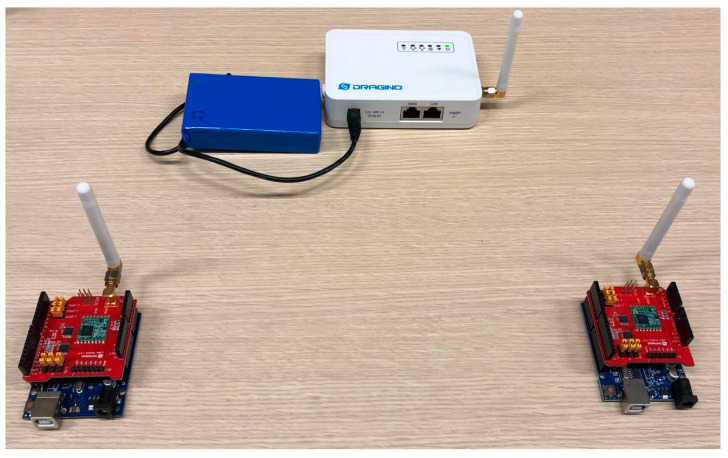
Two LoRa Radios mounted on Audrino UNO with mono-pole antenna and Dragino single channel LoRa gateway.

**Figure 3:**
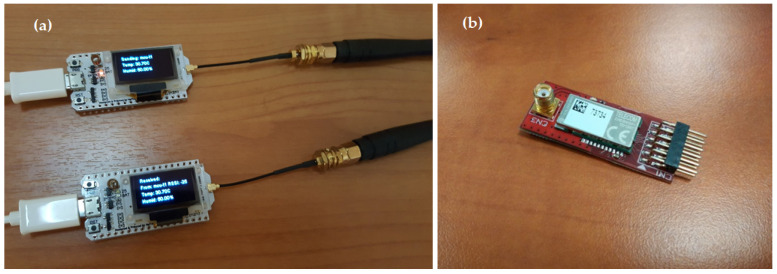
(**a**) Two LoRa devices based on ESP32, (**b**) Sigfox device TD1207.

**Figure 4:**
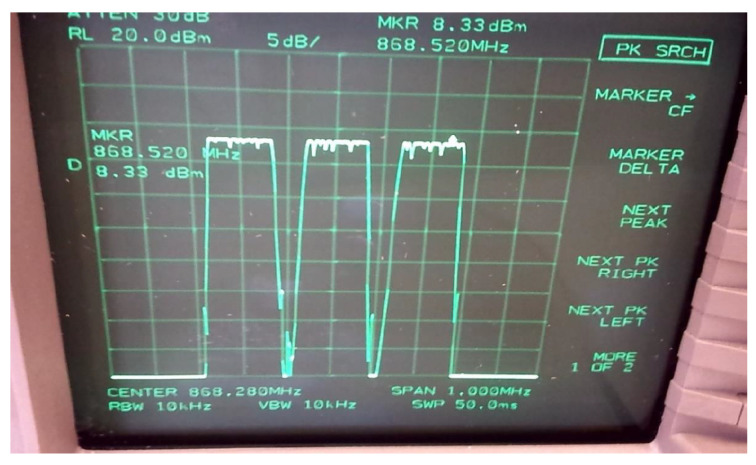
Spectrum analyser output at 868 MHz of LoRaR radios shown in Figure 2.

**Figure 5:**
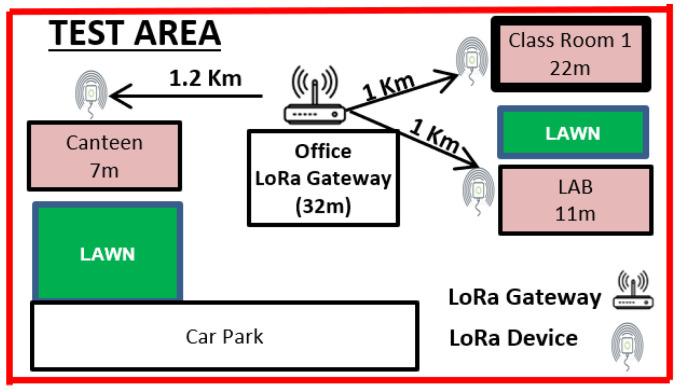
Test setup of LoRa devices as shown in Figure 2 and Figure 3a,b.

Devices used for the tests are shown in Figure 2, Figure 3a,b and spectrum of the same can be seen in Figure 4 while the test setup is shown in Figure 5. It is highlighted that tests for both LoRa and Sigfox were conducted in an outdoor environment with the line of sight (LoS) between devices and gateway. Data generated by the test setup in Figure 5 are shown in Table 3 and the parameters used in Table 4. The traffic generator was programmed with values as given in Table 3 and Table 4, and simulations have been carried out using the data-flow presented in Algorithms 1 and 2 on LoRa/Sigfox devices in multiples of 100 from 0 to 10,000 with random SF and maximum transmission power.

Calculation of RSSI, SNR, and ToA was carried out to prepare data for the traffic generator. LoRa BW was fixed at 250 KHz and packet size at 25 byte. Devices were made to transmit in a 6 h cycle for two days to calculate average values. Further LoRa devices were programmed with different SF and same CR to check collision issues while transmitting 100 up-link messages each to a single channel Dragino gateway, for Sigfox Lime SDR was programmed as gateway using GNU radio.

**Figure 6:**
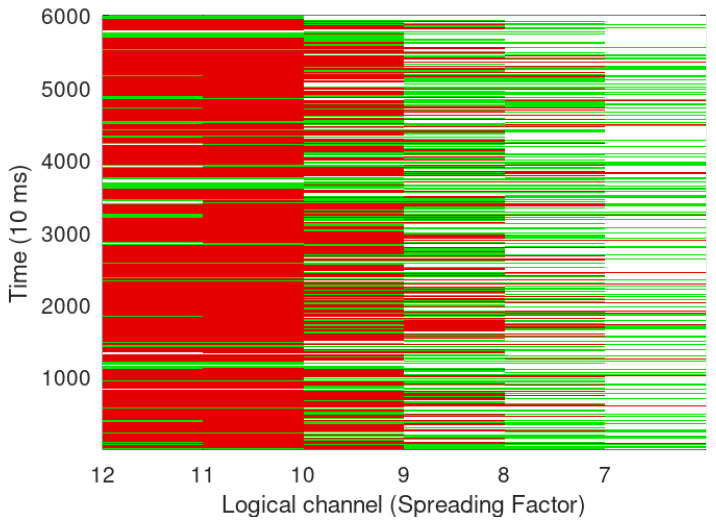
LoRa packet collision simulation with 1000 devices transmitting randomly.

**Figure 7:**
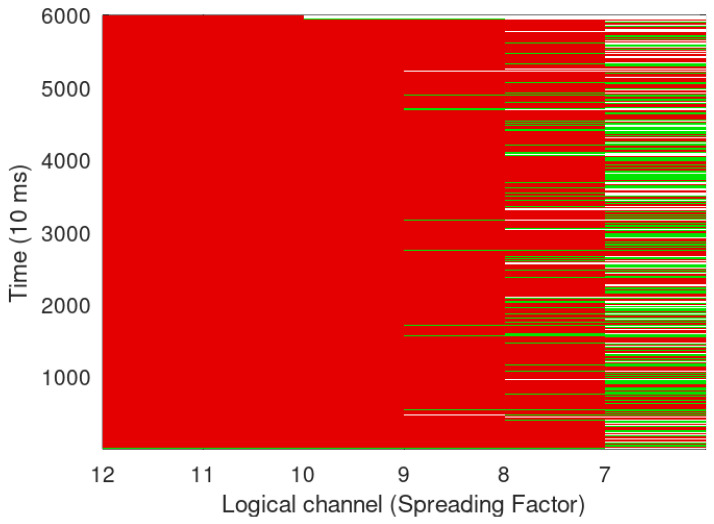
LoRa packet collision simulation with 5000 Devices.

Figure 6 and Figure 7 show LoRa transmission simulation when 1000 and 5000 LoRa devices are transmitting simultaneously, green represents transmission success, while red denotes collision due to the same SF in the use of same-time transmission. Figure 6 and Figure 7 show higher packet loss at higher SF, which is in contravention of LoRa giving higher SNR and higher noise resilience at higher SF. An analysis of the same presents that though the noise robustness is better at higher SF, higher SF also increases the number of chirps and thus increases ToA as calculated in Algorithm 2, which leads to more collisions with more number of re-transmissions.

**Figure 8:**
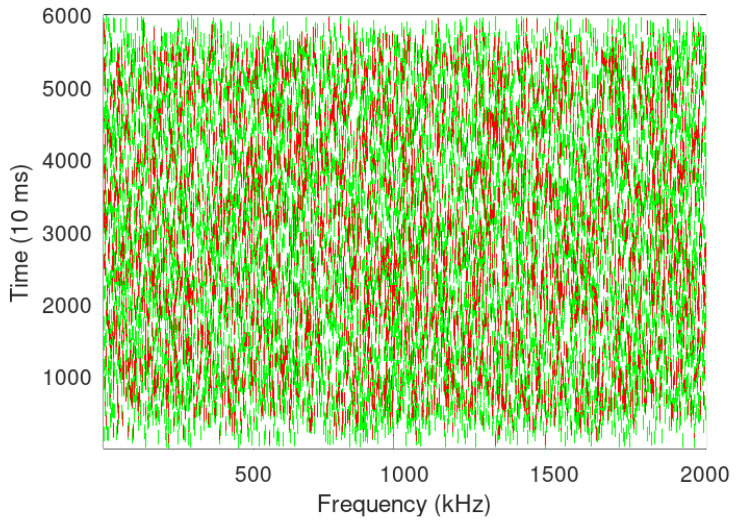
Sigfox simulation for 5000 devices with three gateway.

**Figure 9:**
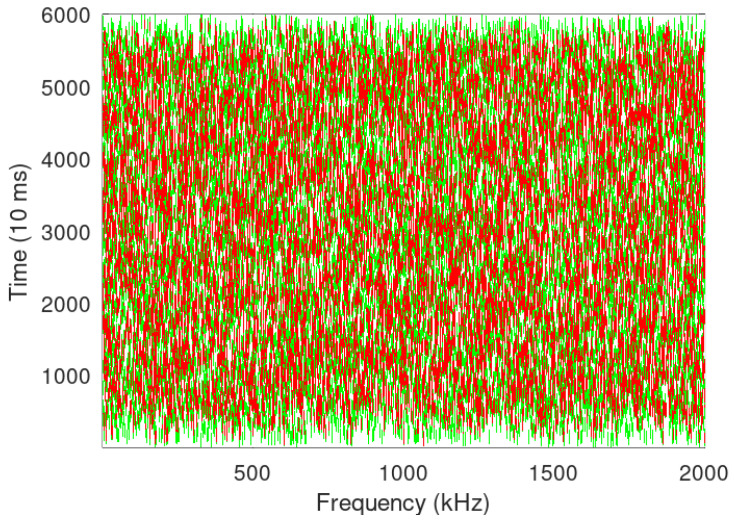
Sigfox simulation for 10000 devices with three gateway.

**Figure 10:**
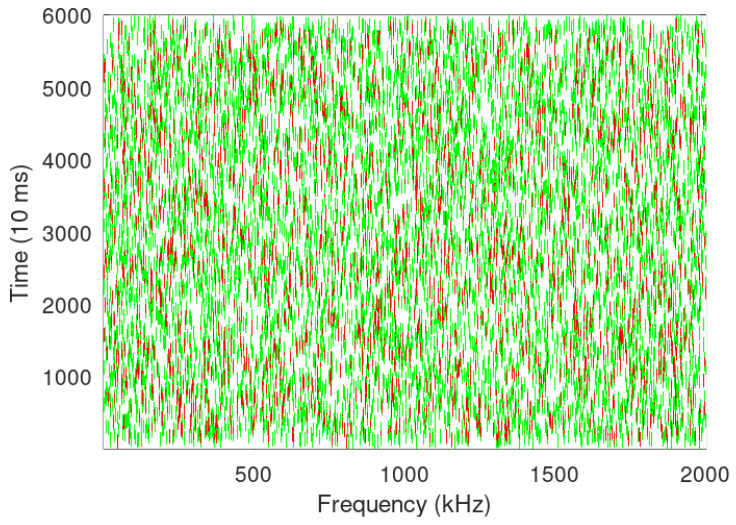
Sigfox simulation for with 10,000 devices with one gateway i.e., not utilizing spatial diversity.

In the Sigfox traffic generator, we started with 50 devices simultaneous operation while keeping only one gateway and then further increasing it to three gateways for spatial diversity. Figure 8 and Figure 9 show 5000 and 10,000 Sigfox devices transmitting simultaneously. PER can be reduced by not utilizing spatial diversity since each Sigfox node transmits to three gateways, and if a message is not acknowledged, the same is re-transmitted, which increases the number of collisions. It can also be seen that LoRa has better performance than Sigfox in congested environment, while Sigfox has a better performance when spatial diversity is not utilised, as seen in Figure 10.

In Figure 6, Figure 7, Figure 8 and Figure 9, it looks that Sigfox has fewer collisions; however, the same is due to Sigfox using half the packet size in comparison to LoRa and also a small BW and data rate. LoRa, on the other hand, does not utilise spatial diversity and thus has a lower PER than Sigfox; in case of the non-receipt of acknowledgement even after two reception windows have passed, LoRa transmits again with incremental SF, thus increasing ToA while sacrificing data rate and contributing to re-transmissions and further collisions, thus increasing devices above a certain limit may end up in choking the entire BW. As Hata propagation model with urban correction factor is taken, −123 dBm is the sensitivity level of a device [15] for SF 7 with an estimated range of 0.80 Km and 1.7 Km in semi-urban setting. Sigfox on the other side, with a sensitivity of −130 dBm and transmitted power of 14 dBm, the estimated range in an urban setting with the Hata model will be up to 3 Km, and with a reduced transmission power of 7 dBm, it will further decrease to 1.9 Km.

**Figure 11:**
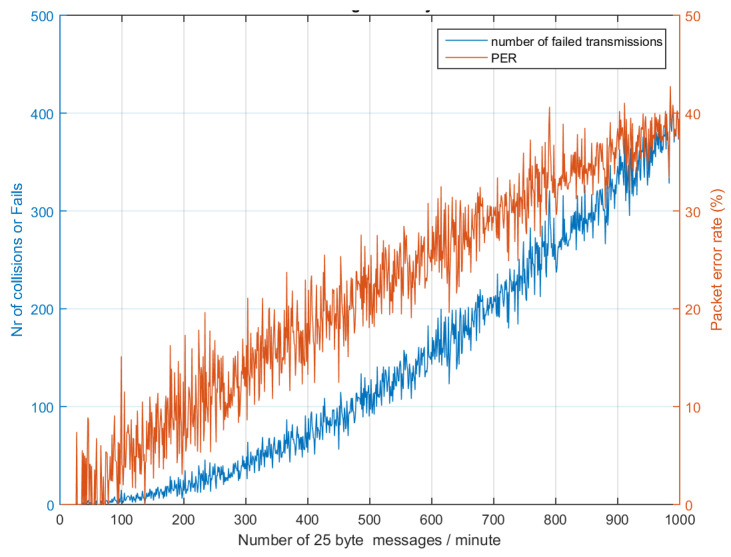
LoRa PER and collision while using SF7, best case (36 ms).

**Figure 12:**
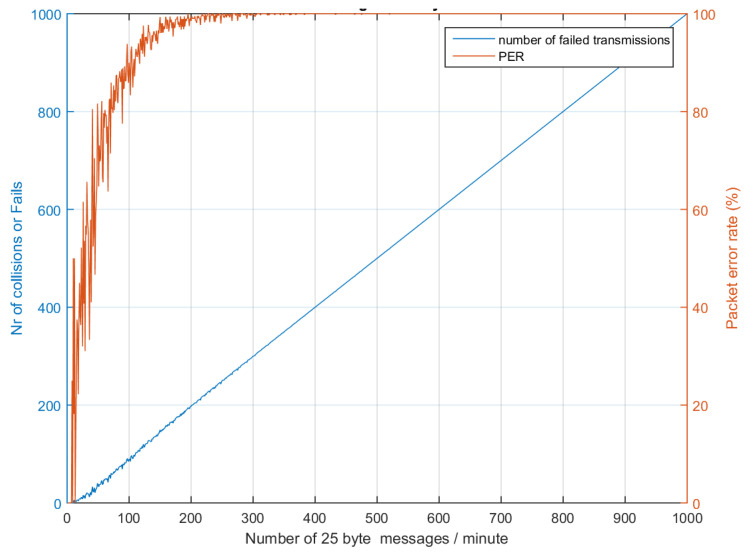
LoRa PER and collision while using SF12, worst case (682 ms).

Table 5 presents the number of collisions calculated during simulation by traffic generator. Equations (23), (26), and (27) have been used to calculate the delivery ratio and cases where no collision occurs. LoRa devices below 100 and Sigfox devices below 200 can safely coexist without collision if the SF and TP assignments are uniform and there are at least two gateways present; the delivery ratio in this case is close to 99.2%, owing to shadow fading and other multipath effects. In case only SF7 is utilized, which has a lower ToA of 36 ms, results can be seen in Figure 11, while if the highest SF12 is used then the PER increases, as shown in Figure 12. It is recommended that PER be maintained below 10% and delivery ratio be kept above 99% for robust communication.

LoRa has a better delivery ratio than Sigfox, as in the case of the former, gateway can dictate transmit power to devices, thus limiting ranges while also increasing the downlink traffic from gateway to device. PER in case of Sigfox reduces by 14% as gateways are reduced from three to one. Table 6 compares this work with previous work carried out on scalability of LoRa and Sigfox.

## 7. Conclusions and Future Scope

LoRa and Sigfox are perfect candidates for long-range IoT requirements with ultra-low-power operation. It emerges that both LoRa and Sigfox are promising technologies that provide different performances in certain conditions and are highly configurable to adjust to the underlying environment. In the first part of the work, design goals and deep insight into LoRa and Sigfox is given, while in the last part, simulations are shown to understand that as the number of devices increases, the number of collisions also increases, which leads to more re-transmissions that may lead to congestion of the network. Results provided in this paper establish that 200 devices of Sigfox and 100 devices of LoRa can coexist without interference beyond the same collisions occur due to the spatial diversity mechanism and re-transmission procedure used by both technologies. Work also takes PER and delivery ratio of packets as the correct figure for estimation of successful transmissions, which takes into account the collision effects, propagation models. The work carried out in this paper can be substantiated in the future by the deployment of a campus network with hundreds of such devices in close proximity to further understand the actual number of collisions through spectrum sensing. In order to increase scalability, it is proposed that the redundancy mechanism of Sigfox be removed and LoRa devices be encouraged to utilize lower SF while they can reach the gateway, and SF 12 or SF 11 be used only in case a device is not able to reach any gateway.

## Figures and Tables

**Table 1:** Technical- and Deployment-Based Specifications of LoRa and Sigfox.

Specifications	LoRa [15,32]	Sigfox [16,33]
Modulation	CSS	DBPSK/GFSK (UNB)
Bandwidth	125/250/500 KHz	100 Hz
Frequency Band	433, 868, 915 MHz	868, 902 MHz
Localization	Available	Not Available
Encryption	AES 128	AES 128
Payload	Upto 255 byte	Upto 12 byte
Adaptive Data Rate	Available	Not Available
Data rate	Upto 50 kbps	600 bps (DL), 100 bps (UL)
Sensitivity	−137 dBm	−129 dBm
Topology	Star of Stars	Star
Devices/Gateway	1 k	1 M
Duty Cycling	1%	1%
MAC	ALOHA	ALOHA
Coding Rate	Available (4)	N/A
Proprietary Aspect	Physical layer	Physical and MAC
Over the air updates	Available	Not Available
Forward Error Correction	Available	Not Available
Message per day	Unlimited	140 (UL)

**Table 2:** LoRa SF with corresponding data rates and SNR values.

Serial No	SF	Symbols	SNR (dB)	Bit Rate (bps)
(a)	7	128	−7.5	5469
(b)	8	256	−10	3125
(c)	9	5125	−12.5	1758
(d)	10	1024	−15	977
(e)	11	2048	−17.5	537
(f)	12	4096	−20	293

**Table 3:** Summary of Test Results.

Parameters	Sigfox	LoRa(SF7)	LoRa(SF12)
Measurements points	3	6	6
Measured parameters	SNR, RSSI	RSSI	RSSI
Maximum SNR	26.96 dB	NA	NA
Minimum SNR	22 dB	NA	NA
Minimum RSSI	−126 dBm	−123 dBm	−121 dBm
Maximum RSSI	−100 dBm	−109 dBm	105 dBm
No of points signal lost	0	0	0
No of available gateway	3	1	1
Standard deviation(SNR)	1.2719	NA	NA
Standard deviation(RSSI)	9.4914	4.42	4.74

**Table 4:** LoRa and Sigfox Simulation Parameters.

Variable Parameters	Value for Simulation
Number of Nodes	50 to 10,000
Spreading Factor	7–12
Code Rate	Fixed 4/8
Bandwidth	Fixed 250 KHz
Message Length	Fixed 25 bytes
Arrival rate ϕ	0.001 s^−1^ bytes
Duty cycle	0.01
Transmission Power	14 dBm
Low data rate optimization (DE)	1
Header (H)	0
Path loss exponent (PLE)	4
Preamble symbols	8
Time on Air (ms)	SF7-41, 8-72, 9-144, 10-289, 11-578, 12-991
Gateway	1, 2

**Table 5:** Collisions as calculated by Traffic Generator.

Nodes	LoRa	Sigfox (3 Gateway)	Sigfox (1 Gateway)
100	8	0	0
500	236	15	2
1000	691	92	14
5000	4614	1892	403
10,000	9617	6526	1240

**Table 6:** Comparative Analysis with Previous Related Work on LoRa and Sigfox.

Work	Year	Nodes	Work Based on
[17]	2016	LoRa; 1600	DER
[18]	2017	LoRa; 10,000	PDR
[20]	2020	Sigfox; 100,000	BER, SNR
[21]	2019	LoRa; 100	PDR, RSSI
[25]	2022	LoRa, Sigfox	PDR, RSSI
Proposed	2024	LoRa-5000, Sigfox 10,000	PER & Delivery Ratio

## Data Availability

Data are contained within the article.
